# Surface Display Technologies for Whole-Cell Biocatalysts: Advances in Optimization Strategies, Food Applications, and Future Perspectives

**DOI:** 10.3390/foods14101803

**Published:** 2025-05-19

**Authors:** Baoyu Zhang, Xing Gao, Yu Zhou, Shengping You, Wei Qi, Mengfan Wang

**Affiliations:** 1 School of Chemical Engineering and Technology, Tianjin University, Tianjin 300350, China; zhangbaoyu@tju.edu.cn (B.Z.); zhouyuy@tju.edu.cn (Y.Z.); ysp@tju.edu.cn (S.Y.); qiwei@tju.edu.cn (W.Q.); 2 School of Life Sciences, Faculty of Medicine, Tianjin University, Tianjin 300072, China; xingao9@tju.edu.cn; 3 Tianjin Key Laboratory of Membrane Science and Desalination Technology, Tianjin 300350, China; 4 State Key Laboratory of Synthetic Biology, Tianjin University, Tianjin 300350, China

**Keywords:** whole-cell biocatalyst, surface display, food applications, immobilization, anchor proteins, passenger proteins

## Abstract

Surface display technology has revolutionized whole-cell biocatalysis by enabling efficient enzyme immobilization on microbial cell surfaces. Compared with traditional enzyme immobilization, this technology has the advantages of high enzyme activity, mild process, simple operation and low cost, which thus has been widely studied and applied in various fields. This review explores the principles, optimization strategies, applications in the food industry, and future prospects. We summarize the membrane and anchor protein structures of common host cells (*Escherichia coli*, *Bacillus subtilis*, and yeast) and discuss cutting-edge optimization approaches, including host strain genetic engineering, rational design of anchor proteins, innovative linker peptide engineering, and precise regulation of signal peptides and promoters, to maximize surface display efficiency. Additionally, we also explore its diverse applications in food processing and manufacturing, additive synthesis, food safety, and other food-related industries (such as animal feed and PET packaging degradation), demonstrating their potential to address key challenges in the food industry. This work bridges fundamental research and industrial applications, offering valuable insights for advancing agricultural and food chemistry.

## 1. Introduction

The advancement of synthetic biology and enzyme engineering has been aided by the accumulation of genomic and protein structural data, with immobilized enzymes emerging as a popular and useful hotspot in this area. The objective of immobilization is to utilize biocatalysts to realize effective biotransformation in biopharmaceuticals, bioenergy, and biomaterial production [1]. Conventional techniques enhance the stability, reusability, and catalytic efficiency through immobilizing enzymes on specific solid carriers (such as chitosan [2], silica-based [3], carbon nanotubes [4], and Fe_3_O_4_ nanoparticles [5], etc. However, these immobilization techniques have some limitations, including complexity in operation, high cost, loss of enzyme activity, and mass transfer resistance. In recent years, cell surface display has emerged as a compelling alternative to conventional immobilization methods.

Cell surface display is a biotechnology with three indispensable components: surface display strain, anchor protein, and passenger protein (enzyme). Passenger proteins are fused with genes encoding specific anchor proteins and secretion signals by means of genetic engineering. Then, the passenger proteins are constructed into expression vectors and introduced into microbial cells so that they can be displayed or immobilized on the surface of the cells [1]. The efficiency of surface display is influenced by the properties of key components in the expression vector, including anchor proteins, passenger proteins, promoters, and signal peptides.

As a promising enzyme immobilization method, cell surface display technology has obvious advantages: Compared with the traditional immobilization method, additional enzyme isolation and purification steps are not needed (Figure S1). For example, the self-transport protein display system of *E. coli* requires only the fusion of the passenger protein gene with the self-transport protein gene to realize the protein display on the bacterial surface [6]. Compared with traditional means such as substance coating, surface-displayed enzymes can directly contact the substrate and reduce mass transfer resistance (Figure S1). Tianyu Li et al. [7] found that surface-displayed enzymes can rapidly initiate catalytic reactions in a short period of time.

Cell surface display improves enzyme stability and activity. Biofilm, as a natural physical barrier, provides some protection for enzymes. And the surface display can avoid the degradation of protease by the integrity of the membrane [8]. As a eukaryotic expression system, yeast surface display system can correctly fold and post-translationally modify proteins, which helps to maintain the natural conformation of the enzyme; the enzyme displayed on the surface of *B. subtilis* spores can maintain better stability under harsh conditions such as high temperature and chemical reagents [9]. The displayed enzyme has higher enzyme activity than the free enzyme [10,11,12].

Cell surface display is also convenient for cell recycling and reuse. The whole-cell biocatalyst constructed based on the surface display system can be recycled by simple centrifugation and other methods to recover the cells at the end of the reaction. It displays on the surface of yeast that it can be reused many times and the catalytic activity can be maintained well in reactions such as biodiesel production [13].

Due to these advantages, the surface display is widely developed, covering phage [14], bacteria [1], fungi [13,15], etc. It can be used in many application scenarios, not only in constructing whole-cell biocatalysts [16,17,18,19], but also in vaccine development [20,21,22,23], biosorption [24,25], and biosensors [26].

In this review, we focus on the advances in cell surface display technology for enzyme immobilization, and attempt to provide a comprehensive overview of the fabrication strategies and the applications of this technology in the food field. In Section 2, we highlight the three most commonly used microbial strains for surface display (*E. coli*, *B. subtilis*, and yeast) and illustrate their membrane structures and corresponding anchor proteins. In Section 3, we discuss in detail the methods to fabricate and optimize the surface display system, with respect to strains, anchor proteins, passenger protein linker peptides, promoters, and signal peptides. In Section 4, we summarize the recent applications of cell surface display in food industries, and finally offer a reasonable future prospect in Section 5.

Compared with the previous review, the new contribution of this review includes (1) providing a systematic summary and comparison of membrane structures and anchor proteins of different strains for surface display, which is beneficial for researchers to select and design a surface display system; (2) offering a comprehensive summary and in-depth analysis of the key factors influencing catalytic efficiency of surface display system, which is helpful for researchers in optimizing the system; and (3) updating the recent achievements of surface display in food applications.

## 2. Commonly Used Strains for Surface Display

Since the size of foreign proteins displayed on the phage surface is quite limited [14], microbial cell surface display systems have been developed [27], and currently, *E. coli*, *B. subtilis*, and yeast have become the most commonly used display strains. In this paper, we summarize the above surface display strains and their commonly used anchoring proteins, including the types of strains used, the size of anchor proteins, and passenger proteins (Table S1).

### 2.1. E. coli

#### 2.1.1. Membrane Structure

**Figure 1:**
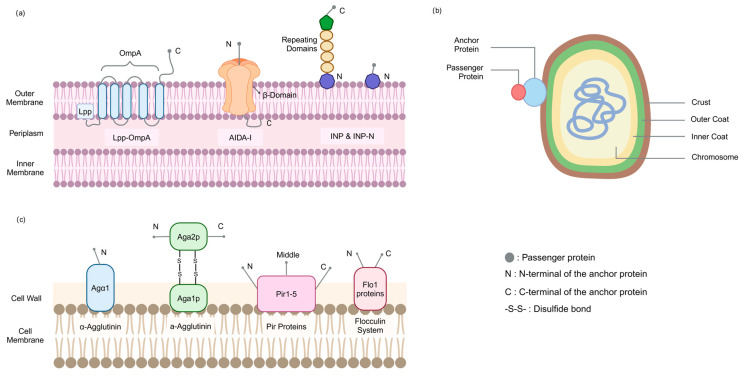
The common strains of surface display systems. (**a**) *E. coli* surface display systems; (**b**) the spore of *B. subtilis* surface display systems; (**c**) yeast surface display systems. Created in https://BioRender.com (accessed on 11 May 2025).

The membrane structure of *E. coli* consists of an inner membrane, an outer membrane, and a periplasmic space. The inner membrane, the cytoplasmic membrane, is a phospholipid bilayer membrane, mainly composed of phospholipids and proteins. The outer membrane is the outer structure of the *E. coli* cell membrane, containing a variety of special components, such as lipopolysaccharide (LPS), which is the endotoxin of Gram-negative bacteria, composed of lipid A, core polysaccharide and O antigen, which plays an important role in maintaining the structural and functional stability of the outer membrane. The outer membrane also contains a variety of proteins, such as β-barrel outer membrane proteins, lipoproteins, and autotransporter proteins (Figure 1a). The periplasmic space is the region between the inner and outer membranes and contains a variety of proteins and enzymes, such as periplasmic proteins involved in protein folding and modification, and proteases that degrade misfolded proteins.

#### 2.1.2. Commonly Used Anchor Proteins

AIDA-I belongs to the classical autotransporter proteins, which are a kind of autotransporter protein found in *E. coli*, containing an N-terminal signal peptide, passenger domain, and C-terminal β-barrel transporter domain. The signal peptide can guide the precursor protein to cross the cytoplasmic membrane via the Sec pathway, and the passenger domain can cross the outer membrane under the action of the β-barrel transporter domain to realize the display of protein on the surface of the cell [28,29].

INP proteins are outer membrane proteins derived from concentrated plant pathogenic bacteria, consisting of approximately 120–180 kDa polypeptides containing three distinguishable structural domains: a relatively hydrophobic N-terminal domain, accounting for about 15% of the total sequence, and containing a membrane-anchoring region linked to mannose-phosphatidylinositol, which is responsible for immobilizing the proteins in the bacterial outer membrane; a C-terminal domain, which is highly hydrophilic and rich in basic amino acid residues, accounting for about 4% of the total sequence; a propionyl-rich domain, which is responsible for immobilizing the proteins in the bacterial outer membrane; and a central repeating domain rich in alanine, glycine, tyrosine, threonine, serine, and leucine, about 80%.

Of these, the N-terminal structural domain appears to be the only prerequisite for successful targeting and surface anchoring, and the central repeat structural domain serves as a spacer region that regulates the distance of the protein from the cell surface. Thus, INP-N, which retains only the N-terminal structural domain, is widely used (Figure 1a) [30,31,32,33].

Outer membrane proteins (OMPs) are important components of Gram-negative bacterial cell membranes, among which β-barrel outer membrane proteins such as OmpA and OmpC are widely used as carriers for surface display [34,35,36,37]. β-Barrel proteins consist of antiparallel β-sheets forming a barrel-like structure that spans the outer membrane. Most OMP-based display systems are only capable of inserting small peptides into surface-exposed loops to maintain stability. Lpp-OmpA is a fusion protein consisting of the signal sequence of Lpp and the first nine residues of the mature protein, as well as residues 46-159 of the OmpA mature protein [38,39]. This fusion protein is anchored to the outer membrane of *E. coli* by the lipid-modified N-terminus of Lpp, which promotes the surface display of larger passenger proteins, but the expression of Lpp-OmpA decreases cell viability [40,41,42].

### 2.2. B. subtilis

#### 2.2.1. Membrane Structure (Spore)

The spore of *B. subtilis* is a complex multilayer structure, the core contains chromosomal DNA, which is maintained in a compact state by small acid-soluble proteins (SASPs), surrounded by the inner membrane, peptidoglycan-rich cortex, and the spore coat in that order [43,44]. The coat consists of about 80 proteins. According to the different locations of formation, the coat proteins are mainly categorized into inner coat proteins (CotD, CotF, OxdD), outer coat proteins (CotA, CotB, CotC, CotE), and crust layer coat proteins (CotG, CotW, CotX, CotY, CotZ) [45] (Figure 1b).

#### 2.2.2. Commonly Used Anchor Proteins

The *Bacillus* coat proteins in spores can be used as anchor proteins. Spores are synthesized in the cytoplasm of the bacterium, so the passenger proteins do not need to cross any cell membrane, but only need to be transported to the surface of the spores under the guidance of the signal peptide of the bacillus clathrin itself. Moreover, molecular chaperones in the cytoplasm of *B. subtilis* can appropriately facilitate the secretion and expression of exogenous proteins, which in turn have a high surface display efficiency. The outer and curst coat proteins have been widely used in the construction of surface display systems, while the inner coat proteins are not suitable for the display of exogenous proteins. CotB, CotC, and CotG are the most commonly used anchor proteins [20,21,22,23,24,25,26,46,47,48].

CotB is the earliest anchoring protein used [49], and its C-terminus consists of three serine-rich repeats, with serine residues accounting for more than half of the C-terminus, making it strongly hydrophilic [50]. Its modification requires the participation of CotG and CotH. CotG interacts directly with CotB, and CotH or its regulated proteins prevent CotG from being hydrolyzed by proteases, indirectly regulating CotB [51].

CotC is an abundant 66-amino acid protein that assembles in the spore shell in various forms, including a 12 kDa monomer, a 21 kDa homodimer, etc. [52]. Its assembly requires CotH and CotE expression, and CotH or CotH-dependent factors prevent CotC from being degraded in the mother cell, thus enabling its assembly in the spore shell [53].

CotG is a 24 kDa protein regulated by the mother cell RNA polymerase σ^K^ and the transcriptional regulator GerE, and GerR can indirectly regulate the expression of CotG through the activation of SpoVIF [54]. CotG is assembled on the spore surface mainly as 32 kDa and 36 kDa proteins [50], and its assembly also requires the expression of CotH, which protects CotG from being hydrolyzed by proteases prior to the formation of spores. CotH protects CotG from being hydrolyzed by proteases before spore formation, which is essential for CotG formation and assembly [9].

### 2.3. Yeast Cells

#### 2.3.1. Membrane Structure

The yeast cell wall is divided into two layers, the inner and outer layers, with a thickness of about 200 nm, accounting for 15–30% of the dry weight of the trophic cells of *Saccharomyces cerevisiae* [55]. The inner layer is mainly composed of β-1,3-glucan, β-1,6-glucan, and chitin (about 50–60% of the dry weight of the cell wall), which provide mechanical strength to the cell wall. The outer layer consists mainly of highly glycosylated mannoproteins, also known as cell wall proteins (CWPs, about 50% of the dry weight of the cell wall), and most of these CWPs are covalently linked to the dextran network, which is involved in processes such as intercellular recognition (Figure 1c) [56,57].

#### 2.3.2. Commonly Used Anchor Proteins

Some cell wall proteins have been used as anchoring proteins for yeast cell surface display. Depending on the mode of binding to the cell wall these proteins can be categorized into three groups: glycosylphosphatidylinositol cell wall proteins (GPI-CWPs) are anchored to the cell surface by glycosylphosphatidylinositol (GPI) [55]; Pir-CWPs are with one or more internal repeat sequences [55]; and flocculating proteins are primarily involved in flocculation [55].

GPI-CWPs have an N-terminal signal peptide that guides the protein into the endoplasmic reticulum, a C-terminal hydrophobic sequence that is replaced by a GPI anchor in the endoplasmic reticulum (ER), a covalent linkage to β-1,6-glucan via a GPI anchor, and a serine- and threonine-rich spacer region between the amino- and carboxy-terminal region [58]. The anchor proteins Sag1 (Agα1), Aga1-Aga2, and Sed1 commonly used in the yeast system are GPI-CWPs. Sag1 (Agα1) is the anchoring unit of the α-Agglutinin system, which is covalently linked to dextran in the cell wall via the C-terminal GPI anchoring attachment signal. The N-terminal secretion signaling region is used for transport to the cell surface and is usually demonstrated at the N-terminal end of the Agα1 destination proteins [59,60]. Aga1-Aga2 is the anchoring unit of a-agglutinin. Aga1 and Aga2 are interconnected by two disulfide bonds. The Aga1 subunit is the anchoring component of the system and is attached to the cell wall via GPI. The Aga2 subunit acts as an adhesion element and connects to the passenger proteins either via the C-terminus or the N-terminus [60,61].

Members of the Pir-CWPs family include Pir1 (CCW6), Pir2 (Hsp150), Pir3 (CCW8), Pir4 (Cis3, CCW5, CCW11) and Pir5 [62]. These proteins consist of an N-terminal signal peptide, a serine protease Kex2 recognition site, a tandem repeat region containing multiple tandem repeats consisting of eight highly conserved amino acids (S, Q, D, G, Q, Q, A, and T), and a carboxy-terminal region. Heterologous target proteins can be fused to the N-terminal or C-terminal end of Pir proteins. In C-terminal fusion, the target protein is anchored to the cell surface with the help of the N-terminal repeat sequence region of Pir proteins; in N-terminal fusion, the target protein is displayed on the cell surface through the formation of disulfide bonds with the cell wall components. Pir4 is also capable of insertion fusion [63,64,65].

Flo1 is a lectin-like protein, which mainly consists of an N-terminal flocculating domain, a secretion signal domain, and a GPI-anchored attachment signal. The flocculating domain near the N-terminal structural domain of Flo1 can noncovalently attach to cell wall components such as mannan carbohydrates, and the heterologous target proteins can be displayed on the surface of the yeast cells by fusion with the N-terminal end of Flo1’s flocculating domain or with the C-terminal end of the GPI-anchored attachment signal [66,67].

## 3. Strategies to Enhance the Catalytic Effect of Whole Cells in Surface Display

**Figure 2:**
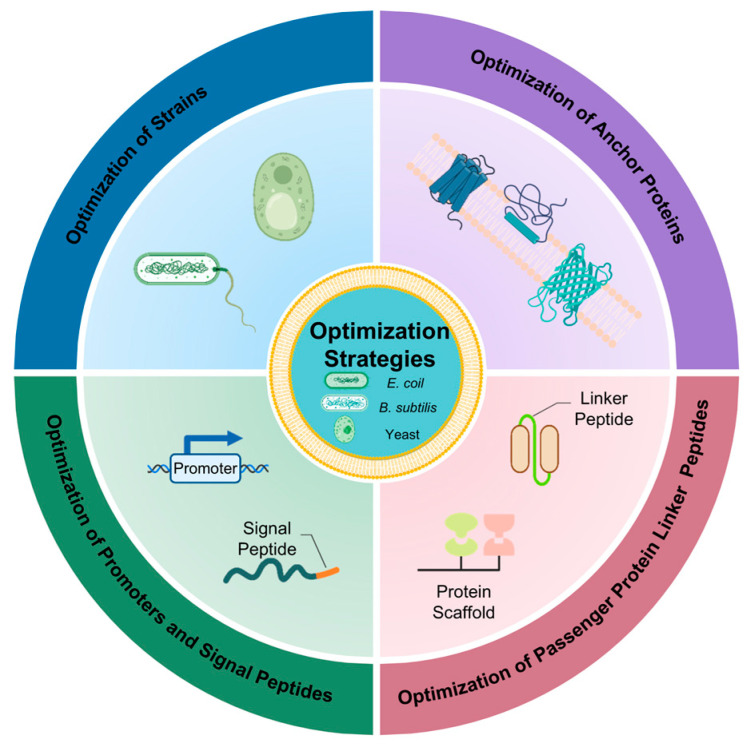
The optimization strategies of surface display systems. Created in https://BioRender.com (accessed on 11 May 2025).

The potential of surface display’s use is determined by its catalytic efficiency. The specific methods to increase the effectiveness of surface display from strains genetic modification, anchor proteins optimization, linker peptides rational selection, and promoters and signal peptides regulation mechanisms are described in depth in this review (Figure 2).

### 3.1. Optimization of Strains

**Figure 3:**
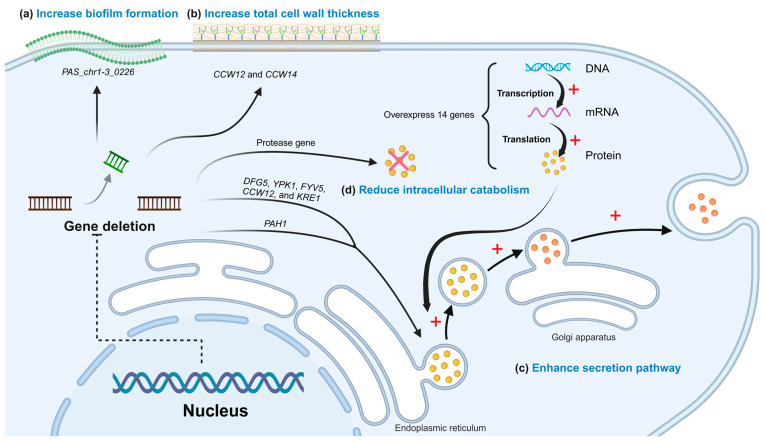
The optimization strategies of Strains. (**a**) The strategies of increasing biofilm formation; (**b**) the strategies of increasing cell wall formation; (**c**) the strategies of enhancing secretion pathway; (**d**) the strategies of reducing intracellular catabolism. + for facilitation, × for decomposition. Created in https://BioRender.com (accessed on 11 May 2025).

Knocking out strain-specific genes to promote cell biofilm and wall formation has proven to be an effective strategy for enhancing surface display efficiency. Tianpeng Chen et al. [18] deleted the biofilm formation gene *PAS_chr1-3_0226* in *S. cerevisiae* GS115 (Figure 3a), which increased biofilm formation by 56% in the knockout strain (∆0226) compared to the wild-type (WT) strain. The ∆0226 strain exhibited a rough surface with biofilm matrix-like material and an enlarged specific surface area. When three anchor proteins (Pir1p, Aga2p, and Flo1p) were employed to display β-galactosidase on the ∆0226 strain surface, the Pir1p anchor protein achieved the highest enzyme activity of 5125 U/g, significantly exceeding that of other recombinant and wild-type strains. Similarly, Kentaro Inokuma et al. [68] demonstrated that simultaneous knockdown of the *CCW12* and *CCW14* genes (Figure 3b), involved in cell wall organization in *S. cerevisiae* BY4741, increased total cell wall thickness and enhanced surface-displayed β-glucosidase (BGL) activity by 1.4-fold compared to the control strain. These findings collectively indicate that cell wall optimization can substantially enhance the surface display of passenger proteins.

Optimizing the secretion pathway represents another effective approach to enhance surface display efficiency. Cell wall biosynthesis can be tightly linked to the secretory pathway by regulation of all involved processes. Nanzhu Chen et al. [69] conducted a comprehensive study of 79 cell wall biosynthesis gene knockouts in *S. cerevisiae* and found that deleting *DFG5*, *YPK1*, *FYV5*, *CCW12*, and *KRE1* genes significantly improved the surface display efficiency of β-glucosidase (BGL1) (Figure 3c). Notably, the double knockout of *FYV5* and *CCW12* showed the most significant effect, with BGL1 activity increasing by 7.99-fold. In addition, Shuo Yang et al. [70] overexpressed 14 genes associated with cell polarization in *S. cerevisiae* and observed that overexpression of the *BUD1* gene alone increased α-amylase surface display by 56%, while co-overexpression of *BUD1* and *CDC42* further enhanced it by 100% (Figure 3c). Likewise, Pamela B Besada-Lombana et al. [71] knockdown of the *PAH1* gene in *S. cerevisiae* resulted in impaired fat droplet formation (Figure 3c), altered fatty acid metabolism pathways, directed lipid flow towards membrane precursor synthesis, and promoted endoplasmic reticulum/nuclear membrane proliferation. These changes enhanced translocation through the secretory pathway, leading to increased membrane protein expression. In their study, *PAH1* knockdown resulted in a 4.2-fold increase in β-glucosidase (BglI) expression compared to the wild-type strain.

Protease activity secreted by host strains can significantly impair the stability and activity of surface-displayed passenger proteins. Zhansheng Li et al. [72] found that the above phenomenon occurs when *P. pastoris* displays RML. By utilizing a protease-deficient *P. pastoris* PichiaPink™ strain as the host (Figure 3d), RML activity reached 121.72 U/g, representing a 46.7% increase compared to the protease-producing recombinant strain.

In summary, structural modifications of host strains, optimization of secretion pathways, and the use of protease-deficient strains collectively contribute to improving the whole-cell biocatalytic efficiency of surface-displayed enzymes.

### 3.2. Optimization of Anchor Proteins

The length of anchor proteins is a critical factor influencing protein surface display, as it significantly impacts display efficiency (Table 1). Lihai Fan et al. [73] investigated the use of ice nucleation protein (INP) variants of different lengths as anchor proteins for displaying carbonic anhydrase (CA). Their results revealed that the shortest fusion protein, INP-N, exhibited the highest expression level and enzymatic activity, suggesting that shorter anchor proteins are advantageous for improving target protein display efficiency in this system. Similarly, Mee-Jung Han et al. [74] employed a C-terminal truncation strategy based on the predicted outer membrane topology of the YiaT anchor protein. Truncations at R181 and R232 in the fourth and fifth extracellular loops resulted in YiaTR181 and YiaTR232 variants with lipase activities approximately 10-fold and 20-fold higher, respectively, compared to commonly used anchor proteins FadL and OprF. Further studies by the same group involved truncating six C-terminal sites (V140, V176, K179, V226, V232, and K234) of the MipA gene, with the MV140 variant demonstrating the highest lipase activity, comparable to that of YiaTR232 [75]. In another study, Xiaoyu Yang et al. [76] addressed the low display efficiency of the complex, structured a-agglutinin system by reconstructing it. They used an innovative strategy to reconstruct the a-agglutinin system by replacing the original Aga1p-Aga2p complex with Aga1p, which contains only the GPI domain. This approach resulted in an almost doubling of the display efficiency and a 39% increase in the activity of the reporter protein BGL. Tea Martinić Cezar et al. [62] designed 14 Hsp150 (Pir2) fusion proteins through a comprehensive analysis of the Pir protein family’s 3D structure, function, genomic organization, and evolution using computational simulations and machine learning. Truncation studies identified Δ7, a minimal Hsp150-based peptide lacking subunit I and most of subunit II, as an efficient N-terminal anchor for surface display. Additionally, they discovered an Hsp150-derived construct with 2.5-fold higher display efficiency than full-length Hsp150 and a Pir tag (4.5 kDa) with equivalent display efficiency to the full-length protein. Optimizing anchor protein length enhances display efficiency and protein activity by reducing inter-protein interactions, optimizing spatial conformation, and minimizing steric hindrance while avoiding adverse effects on cellular physiology. Truncation sites are typically selected based on protein domain information, outer membrane topology predictions, or functional regions [74]. The construction of anchor protein fusion systems with varying lengths provides a robust platform for identifying optimal anchor protein configurations, offering valuable theoretical and practical insights for advancing protein surface display technology.

The development of novel anchor proteins is essential for improving anchoring performance in microbial surface display systems (Table 1). Apisan Phienluphon et al. [77] proposed a systematic approach to identify novel glycosylphosphatidylinositol-anchored cell wall proteins (GPI-CWPs) suitable for *S. cerevisiae*. Using bioinformatics tools, specifically GPIPlus software, they predicted and screened GPI-CWPs based on key C-terminal features, including a serine/threonine-rich (S/T) region with at least 30% S/T residues, a minimum of 10% threonine (T), a length of at least 130 amino acids, and a contiguous T-rich region of 10 amino acids in the C-terminal sequences (CTSs). From this screening, 790 proteins with potential anchoring functions were identified, and 37 GPI-CWPs from various yeast and fungal species were selected for further evaluation. These proteins were used to display yeast-enhanced green fluorescent protein and BGL, with five GPI-CWPs outperforming the conventional α-agglutinin anchor. Notably, an uncharacterized protein from *Kluyveromyces lactis* exhibited the highest CTS efficiency, achieving a BGL activity of 23.5 U/g cell dry weight, which was 2.8-fold higher than that of α-agglutinin. This study provides a robust framework for the discovery and development of novel anchor proteins, offering valuable insights for constructing efficient surface display platforms.

### 3.3. Optimization of Passenger Protein Linker Peptides

Direct fusion of passenger proteins with anchor proteins often disrupts the spatial conformation of the passenger proteins, leading to reduced or lost activity (Table 2). Generally, the above problem will be solved by incorporating a linker peptide between passenger and anchor proteins. Xiaoqiang Jia et al. [78] demonstrated PbrR, PbrR691, and PbrD proteins associated with the adsorption of lead ions (Pb^2+^) on the surface of *E. coli* BL21. They compared four linker strategies: no-connecting peptide (NL), flexible linker peptide (FL, GGGGS), rigid linker peptide (RL, PAPAP), and rigid helical linker peptide (HL, AEAAAKEAAAKA). The flexible linker peptide FL yielded the highest Pb^2+^ adsorption capacities, with values of 404.4-µmol/g cell dry weight (PbrR-FL), 431.7-µmol/g cell dry weight (PbrR691-FL), and 388.4-µmol/g cell dry weight (PbrD-FL). This improvement was attributed to the flexibility and stability of FL, which facilitated proper folding. Similarly, Zhen Wang et al. [79] optimized linker peptides using flexible linker peptide L1 (GGGGS), rigid linker peptide L2 (GGGEAAAKGGG), and extended flexible linker peptide L3 (GGGGSGGGGS). The extended flexible linker peptide L3 achieved the highest activity, increasing it to 131.2 ± 3.4% compared to the control. For larger passenger proteins, such as the 119 kDa cytochrome P450 BM3 (BM3), conventional anchor proteins face limitations in direct gene fusion, hindering effective surface display [40]. SpyTag/SpyCatcher technology, a post-translational biocoupling method, overcomes this limitation. This system involves the fusion of SpyTag (13 amino acids) and SpyCatcher (113 amino acids) domains with passenger proteins, enabling efficient surface immobilization. Sabrina Gallus et al. [40] successfully applied this technique to display BM3 on the surface of *E. coli*. Additionally, Hao Dong et al. [80] developed LBP2-functionalized biofilm materials as a surface display platform to maximize the display of lipase (Lip181). This approach achieved a loading capacity of 27.90 mg/g wet biofilm material (equivalent to 210.49 mg/g dry biofilm material) and demonstrated higher enzymatic activity compared to the SpyTag/SpyCatcher strategy. These findings highlight the critical role of attachment methods in optimizing surface display systems.

Direct surface display involves the fusion genes of passenger proteins with anchor proteins to enable their presentation on the cell surface. However, this approach is often limited by the strain’s membrane load and display efficiency, typically allowing only one or two passenger proteins to be displayed. To overcome these limitations, protein scaffolds are widely utilized to indirectly anchor multiple passenger proteins to the cell surface, thereby enhancing the number of displayed proteins and optimizing membrane surface utilization (Table 2). For instance, Ce Dong et al. [81] employed ultra-high-affinity IM7/CL7 protein pairs and carbohydrate-binding module (CBM) to assemble three recombinant cellulases (including endoglucanase (EG), exoglucanase (CBH), and BGL) on the cell surface to produce bioethanol. This strategy achieved a maximum ethanol titer of 5.1 g/L. Similarly, Yutong Ye et al. [82] utilized protein scaffolds to co-immobilize lytic polysaccharide monooxy-genase (LPMO), versatile peroxidase (VP), and Lac on the surface of *S. cerevisiae* achieving a world-record ethanol titer of 8.68 g/L. In another study, Lizhu Aer et al. [83] demonstrated the efficient degradation of PET by immobilizing PETase, carboxylesterase TfCa, and the hydrophobic binding module CBM3a using protein scaffolds. Protein scaffolds play a pivotal role in enhancing the efficacy and efficiency of biocatalytic reaction systems, driving advancements in related fields. Their application not only increases the density of displayed enzymes but also optimizes their spatial arrangement, thereby improving catalytic performance.

### 3.4. Optimization of Promoters and Signal Peptides

Signal peptides help to guide protein translocation to the cell surface [84], and strong promoters boost the quantity of protein synthesis by increasing the level of gene transcription, which in turn improves surface display efficiency [70]. Table 3 summarizes the optimization of promoters and signal peptides in surface display systems. These optimizations reveal that the choice of promoters and signal peptides is highly host-specific. These optimizations reveal that the choice of promoters and signal peptides is highly host-specific [85]. The display effect can also be enhanced by altering promoters and signal peptides. Stronger promoters are more favorable for improving surface display efficiency [70]. Tingting Chen et al. [86] performed a single base deletion introduced at the OmpF-77 locus upstream of the transcription start site of the OmpF promoter and introduced a silencing mutation (GCA to GCT) at the Ala130 site upstream of the Protospacer Adjacent Motif. The resulting genome-edited strain ER2566-77d results in substantial constitutive expression of OmpF. The resulting genome-edited strain ER2566-77d results in substantial constitutive expression of OmpF in *E. coli*, providing an effective strategy for epitope display on bacterial surfaces. Similarly, Darius Wen-Shuo Koh et al. [87] used an error-prone PCR to engineer Ag43 signal peptides, resulting in mutants that increased surface display levels by 1.4- to 3-fold. These findings underscore the potential of signal peptide modification to significantly enhance surface display efficiency. Therefore, optimization of promoter and signal peptide is an important way to improve the efficiency of surface display.

## 4. Applications in the Food Industry

**Figure 4:**
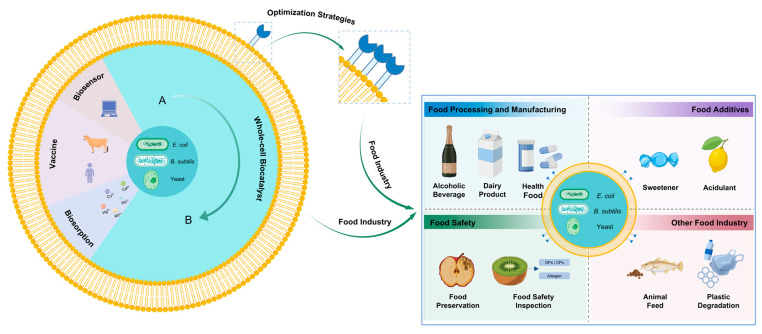
The food application of surface display systems. Created in https://BioRender.com (accessed on 11 May 2025).

Surface display systems have found extensive applications in diverse areas such as whole-cell biocatalysts, vaccine development, biosensors, and biosorption, with whole-cell biocatalysis being the most prominent. The efficiency of surface display has been significantly enhanced through various optimization strategies, which have greatly contributed to advancements in the food sector. This paper provides a comprehensive overview of the recent practical applications of this technology, including food processing and manufacturing, synthesis of food additives, food safety, and other related food applications (Figure 4).

### 4.1. Applications in Food Processing and Manufacturing

#### 4.1.1. Alcoholic Beverages

Surface display technology has gained significant attention in recent years for its potential to enhance the flavor and aroma of wines. *B. subtilis* is a key functional bacterium in the white wine industry, contributing to flavor development. However, its growth is inhibited by high ethanol concentrations, which compromises the flavor profile of white wine. To address this, Yi Yuan et al. [93] displayed *Acetobacter pasteurianus* alcohol dehydrogenase A (adhA) on the surface of *B. subtilis* spores, significantly improving their ethanol tolerance. The recombinant mutant exhibited a viability ratio approximately twice that of the wild-type strain in the presence of 100% ethanol, demonstrating its potential for applications in the alcoholic beverage industry. BGL, a potent glycosidase, plays a crucial role in wine aroma enhancement by hydrolyzing glycoconjugated precursors in grape juice and wine, thereby liberating volatile aromatic compounds. Yang Zhang et al. [88] developed a BGL surface display system on *S. cerevisiae* using the *GPD* promoter and Sed1 anchor protein, achieving a maximum BGL activity of 25.22 ± 0.81 U/g dry cell weight. Furthermore, EGII is shown to hydrolyze grape skin cell wall polysaccharides, disrupting the cell wall structure and facilitating the release of aromatic compounds. Building on this work [88], Rong Huang et al. [94] co-immobilized EGII and BGL on yeast cells separately for the co-fermentation of the two yeasts. A significant increase in the contents of compounds like isoamyl alcohol, phenylethyl alcohol, medium-chain fatty acids, acetate, and ethyl esters than immobilizing BGL alone. This approach enriched the flavor layers of the wines.

Anthocyanins, which contribute to the color of blueberry wine, exhibit limited stability during production. Phenolic acid decarboxylase (PAD) enhances color stability by converting free hydroxycinnamic acids into 4-vinyl derivatives, which react with anthocyanins to form stable pyranoanthocyanidins. However, the low activity of natural PAD in *S. cerevisiae* limits this process. Huaili Deng et al. [95] immobilized PAD on the surface of *S. cerevisiae*, significantly increasing the content of vinylphenolic anthocyanins. Compared to free PAD, the immobilized enzyme produced three new vinylphenolic pyranoanthocyanin products with improved thermal and pH stability, retaining over 88% activity after five cycles. This study demonstrates the feasibility of employing surface display technology to improve color stability during blueberry wine production, offering a novel and effective strategy for enhancing the content of vinylphenolic anthocyanins in fruit wines. This study highlights the potential of surface display technology to improve color stability in fruit wine production, offering a novel strategy for enhancing the content of vinylphenolic anthocyanins.

Surface display technology has also been effectively applied for the detection of catechol in red wine. Zhen Zhang et al. [96] constructed a high-activity whole-cell laccase catalytic system in *E. coli* by fusing a mutant laccase gene (*WlacD*) with three repeats of the N-terminal domain of ice nucleation protein (*inaQ-N*). This system was subsequently immobilized onto a glassy-carbon electrode to construct an electrochemical microbial biosensor for catechol detection. Under optimized pH conditions, the biosensor demonstrated a linear response to catechol concentrations ranging from 0.5 μM to 300.0 μM, with an impressive detection limit of 0.1 μM. The recovery rates for catechol in red wine and tea samples ranged from 97.1% to 103.8%, showing accuracy comparable to high-performance liquid chromatography (HPLC). This study provides a novel and reliable method for the precise quantification of target analytes in wine, highlighting the potential of surface display technology in biosensor applications.

#### 4.1.2. Dairy Products

β-Gal is a key industrial enzyme widely employed as a whole-cell biocatalyst in dairy product development. He Wang et al. [11] successfully displayed β-Gal on the surface of *B. subtilis* spores, achieving a lactulose production yield of 8.8 g/L from a substrate containing 200 g/L lactose and 100 g/L fructose. This yield surpassed that of the free enzyme. Notably, the system retained approximately 30.3% of its initial enzymatic activity after eight consecutive conversion cycles, producing 2.7 g/L lactulose in the final cycle. These results underscore its potential for reuse in industrial applications and offer a promising strategy for efficient lactulose production.

Galactooligosaccharides (GOS), recognized as one of the most important functional oligosaccharide prebiotics [97], have attracted significant interest for their role in developing GOS-enriched dairy products. Tianpeng Chen et al. [18] successfully established a β-Gal surface display system utilizing Pir1p, Aga2p, and Flo1p as anchor proteins. Among these, the Pir1p-based system demonstrated the highest enzymatic activity (5125 U/g) and achieved a GOS conversion rate of 50.3%. Furthermore, this system exhibited exceptional operational stability, sustaining up to 23 consecutive catalytic batches. This study provides innovative insights and methodologies for the industrial-scale production of GOS.

#### 4.1.3. Health Food

Docosahexaenoic acid (DHA), a vital component in health foods, has been efficiently enriched using surface display technology. Li Xu et al. [98] demonstrated *Candida rugosa lipase 1* (CRL LIP1) on the cell wall of *P. pastoris* for DHA enrichment in algae oil. This approach increased the DHA content in glycerides from 40.61% to 50.44%, achieving a 1.24-fold higher yield with a maximum hydrolysis degree of 30.6%.

Phosphatidylserine (PS), valued for its chemical properties and health benefits, is widely used in food, health products, and pharmaceuticals. A promising method for PS synthesis involves the transphosphorylation of phosphatidylcholine (PC) with L-serine catalyzed by phospholipase D (PLD). Baotong Sun et al. [99] employed surface-displayed PLD on *E. coli*, achieving a nearly complete conversion of PC to PS under optimal conditions. The displayed enzyme retained activity for up to three reuse cycles, demonstrating its potential for industrial applications

β-Nicotinamide mononucleotide (β-NMN) has been widely used as a nutraceutical for self-medication. Zhonghui He et al. [100] demonstrated human nicotinamide riboside kinase 2 (NRK-2) on the surface of *S. cerevisiae*. Under optimized conditions, the conversion rate of nicotinamide riboside (NR) to β-NMN reached 98.2%, with a β-NMN concentration of 12.6 g/L, significantly higher than previously reported methods. The whole-cell biocatalyst exhibited excellent pH and thermal stability, with no significant reduction in conversion rate after four reuse cycles. This study provides a safe, stable, and cost-effective biocatalyst for β-NMN production, highlighting its industrial potential.

Gamma-aminobutyric acid (GABA), a key neurotransmitter regulating neuronal excitability and brain functions such as cognition, mood, and sleep, was efficiently produced using surface-displayed glutamic acid decarboxylase (GadB). Sivachandiran Somasundaram et al. [101] displayed GadB from the hyperthermophilic, anaerobic archaeon *Pyrococcus horikoshii* on the surface of *E. coli*. The surface-displayed system demonstrated significantly higher GABA productivity than intracellular expression systems during the initial 12 h of biotransformation. By increasing the reaction temperature, the GABA production rate was enhanced by 12.7-fold. This study offers a novel and efficient approach to the industrial-scale production of GABA.

### 4.2. Applications in Food Additives

#### 4.2.1. Sweeteners

Xylitol, a low-cariogenic and low-glycemic sweetener, offers unique advantages over conventional sugars, making it highly valuable in the food industry [102]. Traditional xylitol production from D-xylose relies on costly and environmentally harmful catalytic hydrogenation processes. As an alternative, biotechnological production using microorganisms such as yeast has emerged as a promising approach. However, the presence of glucose oligomers such as cellobiose in lignocellulosic biomass hydrolysates limits their direct utilization by yeast. To address this, Gregory G Y Guirimand et al. [102] immobilized β-D-glucosidase (AaBGL) on the cell wall of *S. cerevisiae* via the SAG1 anchor domain. This immobilization enabled the degradation of cellobiose into glucose monomers, with glucose being released gradually. As a result, the inhibitory effect of the rapid accumulation of glucose on xylose uptake was avoided, while the cellular energy supply was maintained. This approach ensured the sustainability of the xylose metabolic pathway and provided a stable substrate for xylitol production. Additionally, the cytosolic expression of *Scheffersomyces stipitis* xylose reductase (SsXR) facilitated the conversion of xylose to xylitol, increasing the xylitol/xylose ratio by approximately 2.5-fold. To further enhance xylitol production, multiple transporter proteins were optimized, with the overexpression of the homologous maltose transporter (ScMAL11) increasing xylitol yield by 30%. This study provides a sustainable and efficient technological solution for the industrial production of xylitol.

Isomaltulose, a “generally recognized as safe” (GRAS) ingredient, is widely used as a low-glycemic-index (GI) sweetener in foods due to its less sweetness, non-cariogenic properties, and prebiotic activity [103]. Yijing Zhan et al. [104] demonstrated sucrose isomerase (SIase) on the surface of *B. subtilis* spores using the CotX anchor protein. Under optimal conditions (30 °C, pH 6.0, and 500 g/L sucrose), the system achieved a sucrose conversion rate exceeding 92% within 6 h. Remarkably, the conversion rate remained at 45% after six consecutive batch cycles, demonstrating its potential for reuse. Similarly, Yuan Zheng et al. [105] immobilized pansaccharide sucrose isomerase (pSIase) on the surface of yeast cells achieving a maximum activity of 2910.3 U/g cell dry weight. Using pretreated sugarcane molasses as a substrate at 30 °C, the sucrose conversion rate peaked within 100 min, yielding 184.8 g/L isomaltulose with a conversion efficiency of 92.4%. The system maintained over 85% of its initial conversion rate after nine catalytic cycles. These studies provide efficient and sustainable solutions for the industrial production of isomaltulose.

Trehalose, a widely used sweetener [17], was efficiently produced using a spore surface display system. Hongling Liu et al. [17] displayed a mutant trehalose synthase (V407M/K490L/R680E TreS) on the surface of *B. subtilis* WB800n spores using CotG and CotC as anchor proteins. The combined use of CotG and CotC resulted in higher enzyme activity and stability compared to their individual use. To enhance spore stability and prevent germination during trehalose production, the germination-specific lytic genes *sleB* and *cwlJ* were knocked out from the *B. subtilis* WB800n genome. This system achieved a 74.1% conversion rate of 300 g/L maltose to trehalose within 12 h (350 U/g maltose), with enzyme activity remaining stable after four cycles. These results demonstrate the high efficiency and stability of the spore surface display system for trehalose production, offering a promising approach for industrial applications.

D-Allulose, a novel functional sweetener with anti-obesity properties, is typically synthesized from D-fructose using D-psicose 3-epimerase (DPEase). Weiwei He et al. [106] immobilized DPEase on *B. subtilis* spores using the CotZ anchor protein. Under optimal conditions, the reaction of 500 g/L D-fructose with 30 g/L spores for 12 h produced 85 g/L D-allulose. The system retained 60% of its initial yield after five reuse cycles, demonstrating its potential for industrial-scale D-allulose production.

#### 4.2.2. Acidulants

L (+)-tartaric acid (TA) is a widely used acidulant [107]. Rui Zhou et al. [107] evaluated five anchor proteins (Lpp-OmpA, MipAV140, YiaTR232, InaKN, and InaPbN) for immobilizing *cis*-epoxysuccinate hydrolase (CESH) on the surface of *E. coli*. Among these, the InaPbN-CESH system exhibited the highest whole-cell enzymatic activity. By optimizing the expression conditions, the total activity of the InaPbN-CESHsystem exceeded the total lysate activity of the intracellular CESH[L] overexpression system. Remarkably, the system retained full activity after 15 days of storage at 4 °C, demonstrating its potential for industrial-scale TA production.

D-lactic acid, a biocompatible acidulant that integrates into human metabolic processes without adverse effects, was efficiently produced using engineered *E. coli*. Yuji Aso et al. [108] constructed a recombinant *E. coli* strain by deleting the pyruvate formate-lyase activating enzyme gene (*pflA*) and displaying heterologous BGL on its cell surface. This strain directly utilized cellobiose for D-lactic acid production. In a hollow fiber membrane system, continuous D-lactic acid production was achieved through cyclic cell fermentation. At an optimal cellobiose concentration of 10 g/L, the D-lactic acid yield reached 0.22–0.25 g/L/h, more than three times higher than that of batch fermentation (0.06 ± 0.00 g/L/h). This system offers a novel approach for efficient D-lactic acid production.

Citric acid (CA) is used as an acidulant in the food and beverage industries. XiaoYan Liu et al. [109] immobilized exo-inulinase on the cell surface of the marine-derived yeast *Yarrowia lipolytica* to produce CA. The recombinant yeast produced 77.9 g/L CA and 68.9 g/L CA from inulin in flask-level and 2 L fermentation systems, respectively. This study represents the first report of direct CA production from inulin by engineered *Y. lipolytica*, providing a novel strategy for CA biosynthesis.

#### 4.2.3. Other Food Additives

L-Alpha-glycerylphosphorylcholine (GPC), a compound with significant applications in the food industry, faces challenges in production due to low natural extraction yields, limitations of chemical synthesis, and enzyme-based methods. To address these issues, Longgang Jia et al. [110] investigated a novel cold-adapted phospholipase B in a *P. pastoris* GS115 cell surface display system (dPLB_bv_). This system efficiently produced GPC from oil refinery waste, achieving a yield of 92.7% under optimized conditions. Remarkably, the system retained over 60% of its initial activity after seven catalytic cycles, demonstrating excellent reusability. This study provides a cost-effective and sustainable approach for high-yield GPC production from food processing waste.

Geraniol, a valuable terpenoid widely used in the flavor and fragrance industry, is challenging to produce industrially due to high catalyst costs and environmental concerns. Biaobiao Luo et al. [111] employed a one-pot cascade reaction catalyzed by yeast surface-displayed enzymes for the in vitro biosynthesis of geraniol. Through optimization of catalytic components, cofactor regeneration, and byproduct removal, the system achieved a final geraniol yield of 7.55 mg/L after seven cycles. The high stability and reusability of this surface-displayed enzyme system highlight its potential for scalable industrial production.

### 4.3. Applications in Food Safety

#### 4.3.1. Food Preservation

Pediocin PA-1, a bacteriocin with significant potential in food preservation, faces limitations due to low production yields. To address this, Thu Pham Anh Nguyen et al. [112] anchored it to the surface of *S. cerevisiae*, achieving a high expression level of 4.75 ± 0.75 g dry cell weight per liter of culture in a basic medium. Notably, the recombinant strain exhibited antimicrobial activity against Gram-negative bacteria *Shigella boydii* and *Shigella flexneri*, marking the first report of such activity and broadening the antimicrobial spectrum of Pediocin PA-1. Nevertheless, the recombinant strain exerted no significant inhibitory effect on *Staphylococcus aureus* and *Listeria monocytogenes*. In a related study, Jihwan Chun et al. [113] displayed the endolysin LysSA11, derived from staphylococcal phage SA11, on the surface of *S. cerevisiae*. This system achieved a 5-log reduction of viable *S. aureus* within 3 h and demonstrated superior stability compared to the protein purified from *E. coli*. In addition, Soo Ji Kang et al. [114] utilized the CotG protein to anchor the *Lactobacillus rhamnosus* p75 protein on *B. subtilis* spores, resulting in a 2.0-log reduction in viable *L. monocytogenes* after 6 h of incubation. These studies collectively highlight the potential of surface display technology in developing antimicrobial agents for food preservation.

Chitooligosaccharides (COSs), valuable for food preservation, face production challenges due to limitations in recombinant chitosanase preparation. Qianqian Li et al. [115] constructed a whole-cell catalytic system displaying chitosanase CSN46A on the surface of *E. coli* using one or two ice nucleation protein (InaQ-N) anchor sequences (InaQ-N-CSN46A and 2InaQ-N-CSN46A). The 2InaQ-N-CSN46A system exhibited the highest specific enzyme activity (761.34 ± 0.78 U/g cell dry weight), representing a 45.6% improvement over the InaQ-N-CSN46A system. At a hydrolysis temperature of 60 °C, chitopentaose accumulation reached 77.62%, although enzyme reusability requires further optimization. This system offers a promising strategy for the industrial-scale production of COSs, demonstrating significant application potential.

#### 4.3.2. Food Safety Inspection

In recent years, the detection of pesticide residues and mycotoxins in food using novel biosensors has emerged as a significant research focus. Cholinesterase-based spectrophotometric assays have proven to be an effective approach for the rapid detection of organophosphate pesticides (OPs) and carbamate pesticides (CPs). Jiadong Li et al. [116] employed a codon optimization strategy to enhance the expression of recombinant acetylcholinesterase 2 from *Bombyx mori* (rBmAChE2) in *P. pastoris*. The optimized rBmAChE2 showed a 2.5-fold increase in surface expression on yeast, with an activity of 2280.02 U/g, representing a 2.8-fold improvement over the wild-type enzyme. The enzyme inhibition method based on the optimized rBmAChE2 demonstrated detection limits (0.01–2.69 mg/kg) for 10 tested OPs and CPs that were lower than most thresholds specified in the current Chinese standard method [117] and the maximum residue limits [118]. This method enables more accurate detection of low-concentration pesticide residues, significantly enhancing food safety. Additionally, deoxynivalenol (DON), a prevalent mycotoxin found in cereal crops and their products, poses a serious threat to human health. There is an urgent need for rapid, sensitive, and user-friendly analytical methods for on-site DON detection. Han Yang et al. [119] developed a novel biosensor based on Yeast Surface Display (YSD) technology for the rapid detection of DON. They first constructed a yeast surface display DON-Fab library and subsequently identified four high-sensitivity DON selective yeast Fab@YSD C4 variants using magnetic-activated cell sorting and fluorescence-activated cell sorting. The biosensor achieved a detection limit as low as 0.166 pg/mL, with a linear range of 0.001–132.111 ng/mL, and completed detection within 40 min. This method exhibited high recoveries (93.80–128.00%) and low relative standard deviations (0.49–4.98%) for real samples such as wheat and corn, demonstrating its high sensitivity, specificity, and strong potential for mycotoxin detection in food safety applications

High throughput allergen characterization has traditionally relied on phage display technology. However, this approach is constrained by prokaryotic expression systems, which are associated with issues such as the potential loss of conformational epitopes and the absence of certain translational modifications. These limitations can be surmounted by yeast surface display systems. In this context, Milica Popović et al. [120] proposed a yeast surface display of the kiwifruit allergen actinidin (Act d 1) as a model system to characterize plant-derived food allergens. This innovative approach offers a robust platform for studying food allergens, enhancing the accuracy of immunodiagnostic and immunotherapeutic methods. Furthermore, it provides critical technical support for advancing research in the detection, diagnosis, and treatment of food allergies, paving the way for improved food safety and allergen management strategies.

### 4.4. Applications in Other Food Industry

#### 4.4.1. Animal Feed

Surface display technology holds significant promise in the field of animal feed, offering potential improvements in feed quality, animal performance, and overall health. Its broad application prospects are particularly relevant in addressing challenges such as low phosphorus utilization and environmental pollution caused by the inability of monogastric animals to effectively metabolize phytate due to the lack of phytase in their digestive systems. Patricia L A Muñoz-Muñoz et al. [121] employed surface engineering to modify *E coli*, enabling the display of phytase on its surface. This engineered strain was utilized as a feed additive, effectively mitigating the phytate issue in monogastric animal feed, promoting animal growth, and reducing the environmental impact of farming practices. At the same time, the negative impact of farming on the environment is reduced. Similarly, Chanjuan Liu et al. [122] anchored xylanase from Lentinula edodes (sdLeXyn) to the surface of *P. pastoris*. This system efficiently hydrolyzes xylan in wheat, enhancing feed digestion and absorption in animals. Additionally, sdLeXyn exhibits resistance to pepsin degradation, further enhancing its stability and effectiveness in animal feed applications. These properties underscore the potential of sdLeXyn as a feed additive to improve the utilization of wheat in poultry production, contributing to more sustainable and efficient animal farming practices.

#### 4.4.2. Plastic Degradation

The application of surface display technology has significantly advanced the degradation of PET food packaging, offering a promising solution to environmental challenges. Zhuozhi Chen et al. [10] constructed a whole-cell biocatalyst by displaying PETase on the surface of *S. cerevisiae* cells. Under optimized conditions, this biocatalyst demonstrated a 36-fold increase in degradation efficiency for high crystallinity PET compared to purified PETase. Similarly, Jiayu Hu et al. [12] constructed an *E. coli* surface co-display system incorporating cp52k, mfp-3 strong adhesion protein and Fast-PETase. Notably, the *E. coli* cells co-expressed with mfp-3 and Fast-PETase achieved the highest degradation rate reported to date, degrading over 15% of amorphous PET within 24 h. Wei Han [123] further enhanced PET degradation by developing a dual-enzyme display system featuring Fast-PETase and MHETase, which demonstrated superior degradation efficiency. In a related study, Lizhu Aer et al. [83] utilized a protein scaffold to construct a dual-enzyme system comprising PETase and carboxylesterase TfCa, achieving a PET powder degradation product release of 11.56 ± 0.64 mM over 7 days. These advancements underscore the potential of surface display technology as an innovative and effective approach to PET degradation, contributing to sustainable waste management solutions.

## 5. Future Prospects

Surface display, as a novel technology for enzyme immobilization, has garnered significant attention in recent years due to its high catalytic activity, mild and easy preparation, and broad applications. The biosafety of whole-cell biocatalysts outstands their application in the food industry. However, there are still some challenges for the development of technology, for example, not every industrial strain is amenable to surface display, which means that not all enzymes can be immobilized through this approach. Even for the same strain, the optimal conditions for surface display frequently differ according to the specific target enzyme, thus posing a challenge to the general applicability of the technical process. Furthermore, cells are usually sensitive to strong acids/bases, high temperatures and organic solvents, which restricts their use under stringent reaction conditions.

To circumvent these limitations, it is necessary to get insight into the molecular mechanisms that govern the surface display efficiency and enzyme activity. This involves examining how distinct signal peptides influence the amount of displayed enzyme, the interaction between anchor protein and target enzyme, and the orientation of anchor protein on the cell membrane. In addition, to expand the application horizons of surface-displayed immobilized enzymes, it is required to engineer surface-display host strains that exhibit tolerance to heat, alkalis, and salts. It is anticipated that with the ongoing evolution of synthetic biology theories and technologies, surface display technology will play an increasingly significant role in various fields.

## Figures and Tables

**Table 1:** Optimization of anchor proteins strategies and effects.

Optimization Strategies	Strains	Anchor Proteins	Optimization Methods	Results	References
Truncation of existing anchor proteins	*E. coil* BL21(DE3)	INP	Construction of INP-N (22 KDa) and INP-NC (33 KDa) by truncating INP (114 KDa)	INP-N showed the highest expression level and enzymatic activity.	[73]
	*E. coli* XL10-Gold	YiaT	Truncations at R181 and R232 in the fourth and fifth extracellular loops	The lipase activities of YiaTR181 and YiaTR232 were approximately 10-fold and 20-fold higher, respectively, compared to FadL and OprF.	[74]
	*E. coli* XL10-Gold	MipA	Truncating six C-terminal sites V140, V176, K179, V226, V232, and K234	MV140 variant had the highest lipase activity, comparable to that of YiaTR232.	[75]
	*S. cerevisiae* strain CEN.PK102-5B	Aga1p-Aga2p	Replacing the Aga1p-Aga2p complex with one subunit (Aga1p)	BGL enzyme activity increased by 39%.	[76]
	*S. cerevisiae* BY 4741	Pir protein family	Designing 14 S. cerevisiae Hsp150 (Pir2)-based fusion proteins by machine-learning strategies	The display efficiency of Hsp150-derived constructs was 2.5-fold higher than that of full-length Hsp150.	[62]
Discovery of novel anchor proteins	*S. cerevisiae* BY4743	GPI-CWPs	Designing 37 GPI-CWPs through prediction of GPI-CWPs by GPIPlus and specific feature extraction using Biopython (www.biopython.org)	Among them, five GPI-CWPs outperformed the conventional α-agglutinin anchor.	[77]

**Table 2:** Optimization of passenger protein linker peptides strategies and effects.

Amount of Enzyme	Strains	Optimization Methods	Results	Advantages	Limitations	References
Single-enzyme	*E. coli* BL21 (DE3)	Using four direct fusion methods:(no-connecting peptide (NL), flexible linker peptide (FL, GGGGS), rigid linker peptide (RL, PAPAP), and rigid helical linker peptide (HL, AEAAAKEAAAKA))	The flexible linker peptide FL yielded the highest Pb^2+^ adsorption capacities.	Direct fusion of single passenger proteins: simple and low cost of operation.	Difficult to demonstrate large molecular weight proteins as well as multiple proteins.	[78]
	*B. subtilis* DB 403	Using three direct fusion methods: L1 (GGGGS), L2 (GGGEAAAKGGG), L3 (GGGGSGGGGS)	The extended flexible linker peptide L3 achieved the highest activity.			[79]
	*E. coli* BL21 (DE3)	Using SpyTag/SpyCatcher technology	The most successfully displayed larger passenger protein BM3 (119 KDa).	Indirect fusion of single passenger proteins: large molecular weight proteins can be demonstrated.	Requires in vitro supplementation of purified proteins, which is costly; low assembly efficiency; difficult to demonstrate multiple proteins.	[40]
	*E. coli* BL21 (DE3)	Constructing LBP2-functionalized biofilm material	This approach achieved higher enzymatic activity compared to the SpyTag/SpyCatcher strategy.			[80]
Multi-enzyme	*P. pastoris* GS115	Harnessing an ultra-high-affinity IM7/CL7 protein pair	This way displayed three recombinant cellulases EG, exoglucanase CBH, and BGL to produce bioethanol (the maximum ethanol titer of 5.1 g/L).	Indirect fusion of multiple passenger proteins: a complex catalytic process that can be synergized with multiple enzymes.	Requires in vitro supplementation of purified proteins, which is costly; low assembly efficiency.	[81]
	*S. cerevisiae* EBY100	Utilizing protein scaffolds	*S. cerevisiae* achieved a world-record ethanol titer of 8.68 g/L.			[82]
	*E. coli* BL21 (DE3)	Utilizing protein scaffolds	Degradation reached 11.56 ± 0.64 mM after 7 days.			[83]

**Table 3:** Optimization of promoter and signal peptide strategies and effects.

Strains	Passenger Proteins	Optimization of Promoters	Optimization of Signal Peptides	Results	References
*S. cerevisiae*	α-Amylase and eGFP	*TPI1* and *TDH3p*	-	The strong promoter *TDH3p* increased surface display activity by 23% and 142% when driving α-amylase expression and eGFP, respectively	[70]
*S. cerevisiae*	BGL	*GPD* and *SED1*	-	*GPD* promoter drove BGL with twice the enzyme activity of the *SED1* promoter	[88]
*E. coli*	Hyaluronidase Hyal1	Rhamnose-dependent promoter (P_rha_) and constitutive promoter	-	Replacement of the constitutive promoter by a P_rha_ and optimization of reaction conditions resulted in a 100-fold increase in Hyal1 activity	[89]
*E. coli*	Hepatitis B virus (HBV) S antigen and human papilloma virus (HPV) L2 protein	Mutants of the OmpF promoter	-	Under the OmpF promoter mutation, the proportion of positive cells reached 99.1% and 91.6% for HBV S antigen cell and HPV L2 protein cell, respectively, which was significantly higher than that of the control group	[86]
*S. cerevisiae*	mRuby2	-	9 pre-signal peptides	Among the 9 pre-signal peptides, the AGA2 pre-signal peptides showed the best effect on mRuby2 secretion and surface display	[90]
*E. coli*	sfGFP	-	29 mutants of the Ag43 signal peptide	These mutants increased the level of surface presentation 1.4- to 3-fold	[87]
*S. cerevisiae*	Anti-hen egg-white lysozyme nanobody	*GAP* and *GAL1*	α Pre-pro sequence derived from *S. cerevisiae* and the glucoamylase secretion signal derived from *Rhizopus oryzae*	*GAP* promoter drove more nanobody display than *GAL1* promoter; α pre-pro sequence more suitable for nanobody display	[91]
*P. pastoris*	Multiple antibodies	*ADH1*, *AOD*, *AOX1*, *ENO1*, and *FLD1* five endogenous *P. pastoris* promoters	α-Mating factor (α-MF), α-MF: Δ57-70 and SUC2	It was determined that the combination of the *FLD1* promoter, and SUC2 signal peptide resulted in up to 25% antibody fragment presentation, and that antibody presentation was at least twice as high with *AOX1* and *FLD1* (methanol-inducible promoter) than with ADH1 (glycerol-inducible promoter), *AOD*, and *ENO1* (constitutive promoter); the three signal peptides were similar in their effects	[85]
*P. pastoris*	BGL and EG	*GAP* and *SPI1*	*S. cerevisiae* alpha-factor and *SPI1* secretion signal	The *SPI1* promoter and *SPI1* secretion signal were approximately 1.3-fold and 2.4-fold higher in cell surface BGL and EG activity than the conventional *GAP* promoter and secretion signal of *S. cerevisiae* alpha-factor	[92]
*E. coli*	Tyrosinase	P_laclvs_ and P_rhaB_	Signal peptide of AIDA-I and signal peptide region from the AT Hemoglobin-binding protease	The construct corresponding to the AIDA-I signal peptide had a higher tyrosinase-specific activity; the P_rhaB_ regulated tyrosinase-specific activity was 50% higher than that of P_laclvs_	[84]

## Data Availability

The original contributions presented in the study are included in the article/Supplementary Materials. Further inquiries can be directed to the corresponding author.

## Supplementary Materials

The following supporting information can be downloaded at: https://www.mdpi.com/article/10.3390/foods14101803/s1, Figure S1: The differences between classical enzyme systems and surface display systems; Table S1: Applications in commonly used anchor proteins. References [10,16,17,18,19,20,21,22,23,24,25,26,32,33,34,35,36,37,107,110,116,124,125,126,127,128,129,130,131,132,133,134,135,136,137] are cited in the Supplementary Materials.
