# Supplementary material for: Surface Display Technologies for Whole-Cell Biocatalysts: Advances in Optimization Strategies, Food Applications, and Future Perspectives
Source: Foods. 2025 May 19;14(10):1803. doi: 10.3390/foods14101803 (PMC12111073; doi:10.3390/foods14101803)
Supplement: Supplementary file 1 [file foods-14-01803-s001.zip › foods-3631088-supplementary.pdf]

Surface Display Technologies for Whole-Cell Biocatalysts: Advances in Optimization Strategies, Food Applications, and Future Perspectives

Baoyu Zhang [1,†], Xing Gao [2,†], Yu Zhou [1], Shengping You [1,3], Wei Qi [1,3] and Mengfan Wang [2,4,*]

[1] School of Chemical Engineering and Technology, Tianjin University, Tianjin 300350, China; zhangbaoyu@tju.edu.cn (B.Z.);zhouyuy@tju.edu.cn (Y.Z.); ysp@tju.edu.cn (S.Y.); qiwei@tju.edu.cn (W.Q.)

[2] School of Life Sciences, Faculty of Medicine, Tianjin University, Tianjin 300072, China; xingao9@tju.edu.cn

[3] Tianjin Key Laboratory of Membrane Science and Desalination Technology, Tianjin 300350, China

[4] State Key Laboratory of Synthetic Biology, Tianjin University, Tianjin 300350, China

* Correspondence: mwang@tju.edu.cn

† These authors contribute equally to this work.

Abstract: Surface display technology has revolutionized whole-cell biocatalysis by enabling efficient enzyme immobilization on microbial cell surfaces. Compared with traditional enzyme immobilization, this technology has the advantages of high enzyme activity, mild process, simple operation and low cost, which thus has been widely studied and applied in various fields. This review explores the principles, optimization strategies, applications in the food industry, and future prospects. We summarize the membrane and anchor protein structures of common host cells (*Escherichia coli*, *Bacillus subtilis*, and yeast) and discuss cutting-edge optimization approaches, including host strain genetic engineering, rational design of anchor proteins, innovative linker peptide engineering, and precise regulation of signal peptides and promoters, to maximize surface display efficiency. Additionally, we also explore the diverse applications of it in food processing and manufacturing, additive synthesis, food safety, and other food-related industry (such as animal feed and PET packaging degradation), demonstrating their potential to address key challenges in the food industry. This work bridges fundamental research and industrial applications, offering valuable insights for advancing agricultural and food chemistry.

Keywords: whole-cell biocatalyst; surface display; food applications; immobilization; anchor proteins; passenger proteins

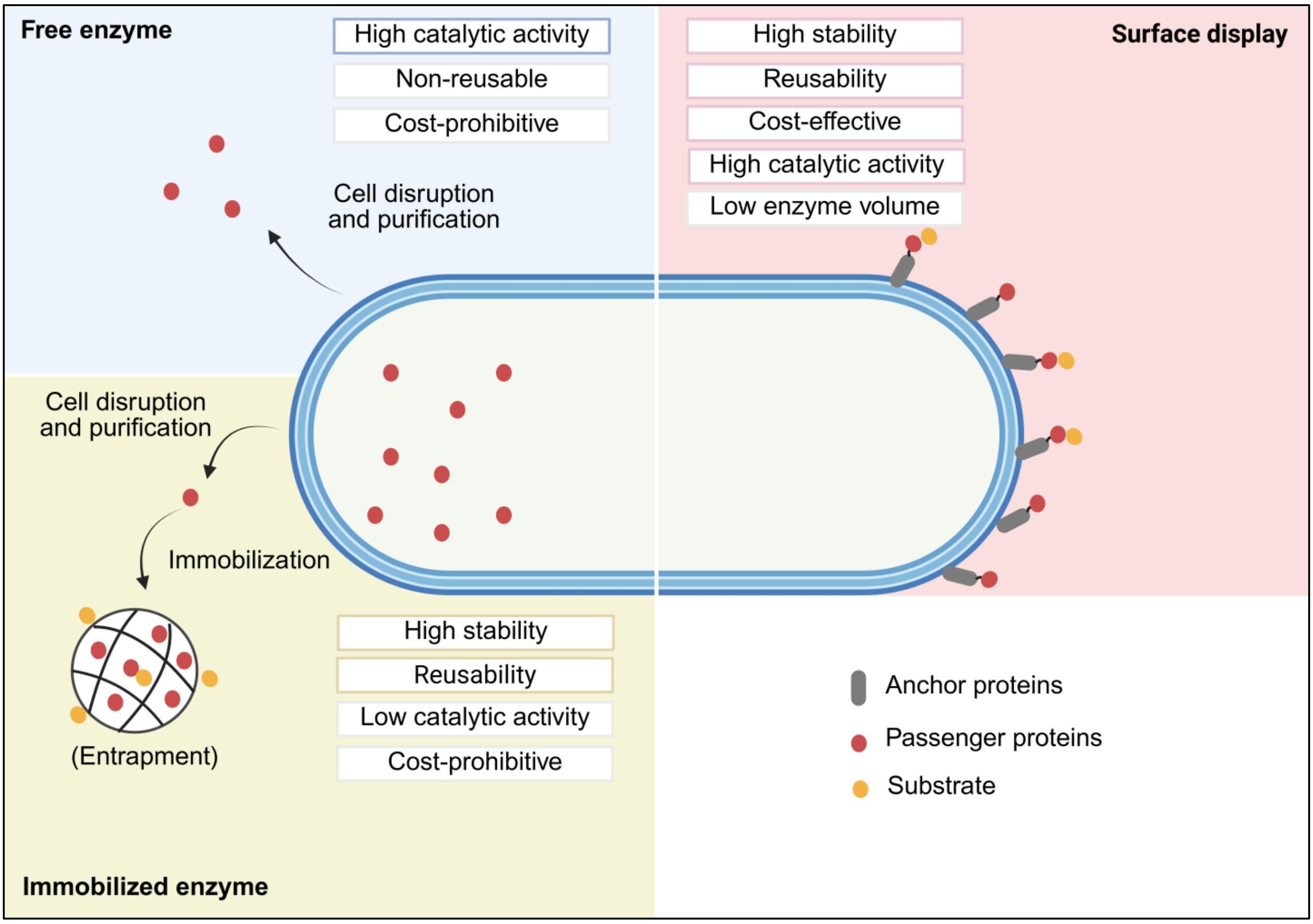

Figure S1. The differences between classical enzyme systems and surface display systems. Created in https://BioRender.com (accessed on 30 June 2025).

Table S1. Applications in commonly used anchor proteins.

Microo-rganism	Strains	Anchor proteins	Source species	Passengers	Results	Applications	Refer-ences
E. coli	*E. coli* BL21 (DE3)	Lpp-OmpA, MipA, YiaT, InaKN and InaPbN	*E. coli* JM109, *E. coli* BL21 (DE3), *E. coli* JM109, *Pseudomonas syringae* and *Pseudomonas borealis*	*Cis*-epoxysuccinic acid hydrolases	Engineered strain displaying CESH[L] via the InaPbN anchoring motif achieved an activity of 1.67 U/OD, with a total activity 18.5-fold higher than that of the intracellular system.	Bioconversion	[1]
	E. coli Nissle 1917	AIDA-I	*E. coli*	Carbohydrate binding domain	Engineered strain with surface-displayed chitin-binding domains demonstrated specific adhesion to Candida albicans hyphae, achieving approximately 2.5% hyphal coverage after 18 hours of co-incubation.	Live biotherapeutic	[2]
	E. coli BL21 (DE3)	AIDA-I and EstA		Klenow fragment of *E. coli* DNA polymerase	The evolved KF variant I709E E710G exhibits a 50.7-fold increase in activity in the synthesis of 2'-O-Me modified DNA.	Directed polymerase evolution	[3]

Table S1. Cont.

Microo-rganism	Strains	Anchor proteins	Source species	Passengers	Results	Applications	Refer-ences
E. coli	*E. coli* BL21 (DE3)	EstA	*Pseudomonas aeruginosa*	Polymer binding peptides	Surface display combined with ultra-high-throughput screening resulted in a 12-fold increase in PP binding load of mutant LCI-CSD-3 (E42V/D45H) in response to surfactant pressure (0.125 mM LAS).	Directed evolution	[4]
	E. coli BL21 (DE3)	InaK-N	*P. syringae*	Cold-active laccase	The engineered strain achieved a surface display efficiency of 88.0%, an enzyme loading of 29.6 U/mg, a degradation efficiency of 48.0% for polyethylene microplastics within 48 h.	Biodegra-dation of polyethylene microplastics	[5]
	E. coli BL21 (DE3)	InaK-N		Erythromycin esterase	The engineered strain ereA completely degraded 50 mg/L erythromycin within 24 h at 25°C, while maintaining 86.7% enzyme activity after 40-day storage and 78.5% activity through seven reuse cycles.	Biodegra-dation of erythromycin	[6]

Table S1. Cont.

Microorganism	Strains	Anchor proteins	Source species	Passengers	Results	Applications	References
E. col	*E. coli* BL21 (DE3)	INP	*P. syringae*	Cr (III) adsorption protein	The engineered M-BL21 system achieved 91.29% Cr(III) removal efficiency, and maintained 68.75% adsorption capacity after five cycles, removing 88.2% Cr(III) from 25 L of tannery wastewater.	Lead Cr (III) adsorption	[7]
	E. coli W-1	INP-N		Methylmercury-binding peptide	Compared to the wild-type strain, the engineered strain removed 96.3% of the 12 μM methylmercury within 2 h and accumulated four times more methylmercury in the cell membrane.	Methylmercury adsorption	[8]
	E. coli BL21 (DE3)	INP-N		Carboxylesterase	The engineered strain retained 120% enzymatic activity after 35 days at 4°C and degraded 85% of 30 mg/L λ-cyhalothrin within 150 min, demonstrating superior stability compared to free enzyme which completely lost activity after 72 hours at 37°C.	Biodegradation	[9]

Table S1. Cont.

Microorganism	Strains	Anchor proteins	Source species	Passengers	Results	Applications	References
E. col	*E. coli* BL21 (DE3)	Lpp-OmpA	*E. coli*	Artificial metalloenzymes	This engineered system successfully degraded lignin surrogate 24 under visible light irradiation, producing 0.28 mM benzaldehyde and 0.23 mM phenol within 24 hours.	Directed evolution	[10]
	E. coli BL21 (DE3)	Lpp-OmpA		*PhotoLanZyme*	The displayed system demonstrated specific activity comparable to purified enzymes, while control cells lacking the scaffold showed minimal background degradation.	Photobio-catalysis	[11]
	E. coli BL21 (DE3)	Lpp-OmpA		Carbonic anhydrase-Sil3K peptide	The biosilica-coated biocatalyst maintained 76% activity at 50°C and demonstrated protease resistance, while retaining 63% catalytic efficiency through ten reuse cycles.	Biochemical conversion	[12]

Table S1. Cont.

Microo-rganism	Strains	Anchor proteins	Source species	Passengers	Results	Applications	Refer-ences
E. col	*E. coli* DH5α	OmpC	*E. coli*	Cobalt binding peptide	The surface-displayed CF2 peptide enabled efficient cobalt adsorption from contaminated wastewater, with recombinant E. coli achieving 1895 μmol/g DCW cobalt uptake at 2 mM concentration.	Cobalt adsorption and degradable dyes	[13]
	E. coli BL21 (DE3)	PgsA	*B. subtilis*	PETase	The engineered strain degraded 94.6% of 1 mM BHET within 60 minutes and released 59 nmol PET monomers from films during 48-hour incubation.	Biodegra-dation of polyethylene terephthalate (PET)	[14]
B. subtilis	*B. subtilis* 168	CotB	*B. subtilis*	HR2-derived peptide	Immunofluoresc ence and flow cytometry confirmed 75% spore surface expression of HR2P-CotB fusion, with western blot showing stable 35 kDa fusion protein.	Vaccine against feline coronavirus	[15]

Table S1. Cont.

Microorganism	Strains	Anchor proteins	Source species	Passengers	Results	Applications	References
B. subtilis	*B. subtilis* 168	CotB	*B. subtilis*	Hemagglutinin–neuraminidase	The surface-displayed HNJD triggered significant upregulation of key cytokines (IL-6, TNF-α, IFN-γ) in intestinal mucosa and enhanced virus-neutralizing antibody titers to 1:320, demonstrating effective dual mucosal and systemic immune activation.	Vaccine against Newcastle disease virus	[16]
	B. subtilis DB403	CotB		Nitrilase	The engineered strain retained 64% activity after 10 reuse cycles and achieved near-complete conversion of 1 M substrates within 24 h, surpassing free nitrilase by 9-16% in cyanocarboxylic acid yields across malononitrile, succinonitrile, and glutaronitrile conversions.	Biochemical conversion	[17]

Table S1. Cont.

Microorganism	Strains	Anchor proteins	Source species	Passengers	Results	Applications	References
B. subtilis	*B. subtilis* 168	CotC	*B. subtilis*	CO-26K equivalent	Oral administration of recombinant spores generated significantly elevated IgG antibodies and cytokines compared to controls.	Vaccine against porcine epidemic diarrhoea virus	[18]
	B. subtilis WB600	CotC		Largemouth bass virus (LMBV)	The CotC-LMBV recombinant spores had stable antigen-presenting ability, and specific IgM levels increased 2.1-fold after inoculation compared to controls.	Vaccine against LMBV	[19]
	B. subtilis DB104	CotE, CotG and CotY		Laccase (Lac)	The engineered strain achieved 48.75% decolorization of Acid Green 25 within 5 hours. When tested against structurally diverse dyes, the recombinant spores demonstrated 18.58% decolorization of Acid Red 18 (azo dye) and 43.20% efficiency for indigo carmine.	Decolorization of Acid Green 25	[20]

Table S1. Cont.

Microorganism	Strains	Anchor proteins	Source species	Passengers	Results	Applications	References
B. subtilis	*B. subtilis* 168	CotG	*B. subtilis*	LGG-derived p75 protein	CotG-p75 upregulated 218 genes, including NF-KB signaling components (NFKBIA, RELB) and chemokines (CXCL1, CXCL8, CCL20), enhancing immune regulation and chemotaxis.	Immunomodulatory analysis	[21]
	B. subtilis WB800N	CotG		Exochitinase	The engineered strain achieved 16.06 U/mL chitinolytic activity, enhanced by Ni^{2+}, Zn^{2+}, and EDTA, while maintaining 62.7% residual activity after four recycling rounds.	Bioconversion	[22]
	B. subtilis WB800N	CotG and CotC		Trehalose synthase	The co-displayed enhanced enzymatic activity to 1,511.6 U/g spores, resulting in 74.1% conversion rate of 300 g/L maltose within 12 hours and maintained 73% activity after four consecutive cycles.	Bioconversion	[23]

Table S1. Cont.

Microo-rganism	Strains	Anchor proteins	Source species	Passengers	Results	Applications	Refer-ences
Yeast	*Pichia pastoris* GS115	Pir1p, Aga2p and Flo1p	*S. cerevisiae*	β-Galactosidase (β-Gal)	The engineered strain achieved 5,125 U/g enzymatic activity with 50.3% galactooligosaccharides conversion efficiency from lactose. Combined with biofilm-immobilized fermentation, the system maintained catalytic stability through 23 consecutive batches.	Bioconversion	[24]
	P. pastoris GS115	α-Agglutinin (AGα1)		Cold-adapted chitosanase	The engineered strain achieved a high yield of 102,415 U/g dry cell weight, exhibited enhanced thermostability (retaining >90% activity at 20–40°C for 5 h) and broader pH tolerance (pH 4–8),and retained 76% activity after six reuse cycles.	Bioconversion	[25]

Table S1. Cont.

Microorganism	Strains	Anchor proteins	Source species	Passengers	Results	Applications	References
Yeast	*P. pastoris* GS115	α-Agglutinin (AGα1)	*S. cerevisiae*	Acetylcholine-sterase	The engineered strain demonstrated 2.8-fold higher catalytic efficiency compared to wild-type displayed enzyme while maintaining equivalent sensitivity to organophosphate and carbamate pesticides.	Pesticide residue screening detection	[26]
	S. cerevisiae EBY100	a-Agglutinin (Aga1/Aga2)		RBDs of Spike protein	The surface-displayed RBD variants of SARS-CoV-2 Spike protein, particularly B.1.617.1, elicited robust humoral responses with neutralizing antibody titers reaching 1:64.	Vaccine against severe acute respiratory syndrome coronavirus-2	[27]
	S. cerevisiae EBY100	a-Agglutinin (Aga1/Aga2)		Glucose dehydrogenase, cholesterol oxidase	The GDH-based biosensor achieved rapid glucose detection (8.5 s response) with a linear range of 20–600 mg/dL (1.4–33.3 mmol/L), while the CHO-based biosensor detected cholesterol within 30 s (2–6 mmol/L linear range).	Biosensors	[28]

Table S1. Cont.

Microorganism	Strains	Anchor proteins	Source species	Passengers	Results	Applications	References
Yeast	*S. cerevisiae* EBY100 and pYD1	a-Agglutinin (Aga1/Aga2)	*S. cerevisiae*	ORF131	The surface-displayed CyHV-3 ORF131 on yeast enabled effective oral antigen delivery to carp midgut cells, triggering mucosal IgT1 response and systemic IgM production.	Vaccine against Cyprinid Herpesvirus 3	[29]
	P. pastoris GS115	Flo1 (FS)		Cold-adapted phospholipase B	The cold-active dPLBbv maintained 92.7% GPC yield at 20°C within 3 hours and retained >60% productivity after 7 catalytic cycles, demonstrating enhanced thermal/pH stability compared to free enzyme.	Biochemical conversion	[30]
	P. pastoris GS115	Flo1p		Laccase	The whole-cell biocatalyst achieved 97.8% BPA degradation (91% efficiency) with 55% activity retention after 4 operational cycles, outperforming free laccase while maintaining thermostability at 70°C for 120 minutes.	Biodegradation of bisphenol A	[31]

Table S1. Cont.

Microorganism	Strains	Anchor proteins	Source species	Passengers	Results	Applications	References
Yeast	*P. pastoris* GS115	Pir1p	*S. cerevisiae*	Rh metal peptides	The engineered strain achieved 75.03% removal (142.11 mg/g) in simulated wastewater, and demonstrated 48.49% adsorption capacity and 65.86% selectivity for Rh(III), while 34.49% desorption efficiency was attained using 2-M HNO_3 coupled with ultrasonic treatment.	Lead Rh (III) adsorption	[32]
	P. pastoris X-33	Pir1		Lipase from *Rhizomucor miehei* (RML)	The engineered strain (10%) achieved 79.1% conversion of soybean fatty acid distillate (SFAD) to ethyl esters within 48 h using. Crosslinking with glutaraldehyde significantly improved operational stability, maintaining 87.9% initial activity after six reuse cycles in solvent-free systems.	Biodiesel production	[33]

Table S1. Cont.

Microorganism	Strains	Anchor proteins	Source species	Passengers	Results	Applications	References
	S. cerevisiae BY4741	Sed1 and Sag1		Enhanced green fluorescent protein (eGFP)	Immunoelectron microscopy revealed Sed1-directed proteins predominantly localized at the outer cell wall (120-150 nm from plasma membrane), while Sag1-anchored enzymes occupied inner layers (40-70 nm depth).	Anchorage position control	[34]
	P. pastoris GS115	GCW21, 51 and 61	*P. pastoris*	PETase	The engineered strain exhibited a 36-fold higher turnover rate compared to purified PETase, maintaining stable activity through seven reuse cycles while degrading commercial PET bottles effectively under optimized conditions.	Biodegradation of PET	[35]

* Strain selection reflects host-organism specialization: *E. coli* BL21(DE3) (protease-deficient, T7 RNA polymerase-integrated) drives industrial biocatalysis via high-yield protein expression; *E. coli* Nissle 1917 leverages probiotic gut colonization for live biotherapeutics; *E. coli* DH5α/JM109 (recA-/lacZΔM15) ensure plasmid stability/cloning fidelity. *B. subtilis* 168 is a model strain with well-characterized sporulation machinery; *B. subtilis* WB600 and *B. subtilis* WB800N are extracellular protease-deficient for stable secretion of heterologous enzymes. *P. pastoris* GS115 contains a methanol-inducible AOX1 promoter system for high-density expression of secreted proteins; *S. cerevisiae* EBY100 contains the GAL1-inducible Aga1/Aga2 system for yeast surface display applications; *S. cerevisiae* BY4741 has a synthetic biology nutrient-deficient marker.

References

1. Zhou, R.; Dong, S.; Feng, Y.; Cui, Q.; Xuan, J. Development of highly efficient whole-cell catalysts of *cis*-epoxysuccinic acid hydrolase by surface display. *Bioresources and Bioprocessing* **2022**, *9*, doi:10.1186/s40643-022-00584-6.
2. Chamas, A.; Svensson, C.-M.; Maneira, C.; Sporniak, M.; Figge, M.T.; Lackner, G. Engineering Adhesion of the Probiotic Strain *Escherichia coli* Nissle to the Fungal Pathogen *Candida albicans*. *ACS Synthetic Biology* **2024**, *13*, 4027-4039, doi:10.1021/acssynbio.4c00466.
3. Chung, M.E.; Goroncy, K.; Kolesnikova, A.; Schönauer, D.; Schwaneberg, U. Display of functional nucleic acid polymerase on *Escherichia coli* surface and its application in directed polymerase evolution. *Biotechnology and Bioengineering* **2020**, *117*, 3699-3711, doi:10.1002/bit.27542.
4. Apitius, L.; Rübsam, K.; Jakesch, C.; Jakob, F.; Schwaneberg, U. Ultrahigh-throughput screening system for directed polymer binding peptide evolution. *Biotechnology and Bioengineering* **2019**, *116*, 1856-1867, doi:10.1002/bit.26990.
5. Zhang, A.; Hou, Y.; Wang, Y.; Wang, Q.; Shan, X.; Liu, J. Highly efficient low-temperature biodegradation of polyethylene microplastics by using cold-active laccase cell-surface display system. *Bioresource Technology* **2023**, *382*, doi:10.1016/j.biortech.2023.129164.
6. Liu, M.; Feng, P.; Kakade, A.; Yang, L.; Chen, G.; Yan, X.; Ni, H.; Liu, P.; Kulshreshtha, S.; Abomohra, A.E.-F.; et al. Reducing residual antibiotic levels in animal feces using intestinal *Escherichia coli* with surface-displayed erythromycin esterase. *Journal of Hazardous Materials* **2020**, *388*, doi:10.1016/j.jhazmat.2020.122032.
7. Wang, J.; Zhao, S.; Ling, Z.; Zhou, T.; Liu, P.; Li, X. Enhanced removal of trivalent chromium from leather wastewater using engineered bacteria immobilized on magnetic pellets. *Science of The Total Environment* **2021**, *775*, doi:10.1016/j.scitotenv.2021.145647.
8. Liu, M.; Lu, X.; Khan, A.; Ling, Z.; Wang, P.; Tang, Y.; Liu, P.; Li, X. Reducing methylmercury accumulation in fish using *Escherichia coli* with surface-displayed methylmercury-binding peptides. *Journal of Hazardous Materials* **2019**, *367*, 35-42, doi:10.1016/j.jhazmat.2018.12.058.
9. Ding, J.; Liu, Y.; Gao, Y.; Zhang, C.; Wang, Y.; Xu, B.; Yang, Y.; Wu, Q.; Huang, Z. Biodegradation of λ-cyhalothrin through cell surface display of bacterial carboxylesterase. *Chemosphere* **2022**, *289*, doi:10.1016/j.chemosphere.2021.133130.
10. Baiyoumy, A.; Vallapurackal, J.; Schwizer, F.; Heinisch, T.; Kardashliev, T.; Held, M.; Panke, S.; Ward, T.R. Directed Evolution of a Surface-Displayed Artificial Allylic Deallylase Relying on a GFP Reporter Protein. *Acs Catalysis* **2021**, *11*, 10705-10712, doi:10.1021/acscatal.1c02405.
11. Klein, A.S.; Leiss-Maier, F.; Mühlhofer, R.; Boesen, B.; Mustafa, G.; Kugler, H.; Zeymer, C. A De Novo Metalloenzyme for Cerium Photoredox Catalysis. *Journal of the American Chemical Society* **2024**, *146*, 25976-25985, doi:10.1021/jacs.4c04618.
12. Abdelhamid, M.A.A.; Son, R.G.; Ki, M.R.; Pack, S.P. Biosilica-coated carbonic anhydrase displayed on *Escherichia coli*: A novel design approach for efficient and stable biocatalyst for CO_2 sequestration. *International Journal of Biological Macromolecules* **2024**, *277*, doi:10.1016/j.ijbiomac.2024.134058.
13. Kumaravel, A.; Selvamani, V.; Sengupta, T.; Gu Kang, S.; Ho Hong, S. Surface engineered recombinant *Escherichia coli* for the potential application of the cobalt contaminated wastewater treatment and the photocatalytic dye degradation. *Bioresource Technology* **2024**, *403*, doi:10.1016/j.biortech.2024.130796.
14. Yamashita, T.; Matsumoto, T.; Yamada, R.; Ogino, H. Display of PETase on the Cell Surface of *Escherichia coli* Using the Anchor Protein PgsA. *Applied Biochemistry and Biotechnology* **2024**, *196*,

5471-5483, doi:10.1007/s12010-023-04837-8.
15. Chen, C.; Li, Y.L.; Lv, F.L.; Xu, L.D.; Huang, Y.W. Surface Display of Peptides Corresponding to the Heptad Repeat 2 Domain of the Feline Enteric Coronavirus Spike Protein on *Bacillus subtilis* Spores Elicits Protective Immune Responses Against Homologous Infection in a Feline Aminopeptidase-N-Transduced Mouse Model. *Frontiers in Immunology* **2022**, *13*, doi:10.3389/fimmu.2022.925922.
16. Li, J.; Yang, M.; Chen, B.; Wang, Z.; Cao, Y.; Yang, Y.; Zhang, M.; Zhang, D.; Ni, X.; Zeng, Y.; et al. Evaluation of the Immunity Responses in Mice to Recombinant *Bacillus subtilis* Displaying Newcastle Disease Virus *HN* Protein Truncations. *Microorganisms* **2024**, *12*, doi:10.3390/microorganisms12030439.
17. Zhong, X.; Yang, S.; Su, X.; Shen, X.; Zhao, W.; Chan, Z. Production of Cyanocarboxylic Acid by *Acidovorax facilis* 72W Nitrilase Displayed on the Spore Surface of *Bacillus subtilis*. *Journal of Microbiology and Biotechnology* **2019**, *29*, 749-757, doi:10.4014/jmb.1901.01030.
18. Tian, Y.; Wang, Z.; Sun, J.; Gu, J.; Xu, X.; Cai, X. Surface display of the COE antigen of porcine epidemic diarrhoea virus on *Bacillus subtilis* spores. *Microbial Biotechnology* **2024**, *17*, doi:10.1111/1751-7915.14518.
19. Wang, Q.; Liang, X.; Ning, Y.; Liu, S.; Liang, Z.; Zhang, Z.; Chen, Y.; Cao, J.; Wang, F.; Lan, L.; et al. Surface display of major capsid protein on *Bacillus subtilis* spores against largemouth bass virus (LMBV) for oral administration. *Fish & Shellfish Immunology* **2023**, *135*, doi:10.1016/j.fsi.2023.108627.
20. Park, J.-H.; Kim, W.; Lee, Y.-S.; Kim, J.-H. Decolorization of Acid Green 25 by Surface Display of CotA laccase on *Bacillus subtilis* spores. *Journal of Microbiology and Biotechnology* **2019**, *29*, 1383-1390, doi:10.4014/jmb.1907.07019.
21. Kang, S.-J.; Jun, J.-S.; Hong, K.-W. Transcriptome Analysis Reveals Immunomodulatory Effect of Spore-Displayed p75 on Human Intestinal Epithelial Caco-2 Cells. *International Journal of Molecular Sciences* **2022**, *23*, doi:10.3390/ijms232314519.
22. Ullah, M.; Xia, Y.; Alshaya, D.S.; Han, J.; Attia, K.A.; Shah, T.A.; Chen, H. Display of Bacterial Exochitanase on *Bacillus subtilis* Spores Improved Enzyme Stability and Recyclability. *Molecules* **2024**, *29*, doi:10.3390/molecules29184302.
23. Liu, H.; Yang, S.; Wang, X.; Wang, T. Production of trehalose with trehalose synthase expressed and displayed on the surface of *Bacillus subtilis* spores. *Microbial Cell Factories* **2019**, *18*, doi:10.1186/s12934-019-1152-7.
24. Chen, T.; Wang, S.; Niu, H.; Yang, G.; Wang, S.; Wang, Y.; Zhou, C.; Yu, B.; Yang, P.; Sun, W.; et al. Biofilm-Based Biocatalysis for Galactooligosaccharides Production by the Surface Display of β-Galactosidase in *Pichia pastoris*. *International Journal of Molecular Sciences* **2023**, *24*, doi:10.3390/ijms24076507.
25. Peng, Y.; Wang, Y.; Liu, X.; Zhou, R.; Liao, X.; Min, Y.; Ma, L.; Wang, Y.; Rao, B. Expression and Surface Display of an Acidic Cold-Active Chitosanase in *Pichia pastoris* Using Multi-Copy Expression and High-Density Cultivation. *Molecules* **2022**, *27*, doi:10.3390/molecules27030800.
26. Li, J.; Xie, X.; Cai, J.; Wang, H.; Yang, J. Enhanced Secretory Expression and Surface Display Level of *Bombyx mori* Acetylcholinesterase 2 by *Pichia pastoris* Based on Codon Optimization Strategy for Pesticides Setection. *Applied Biochemistry and Biotechnology* **2021**, *193*, 3321-3335, doi:10.1007/s12010-021-03597-7.
27. Xing, H.; Zhu, L.; Wang, P.; Zhao, G.; Zhou, Z.; Yang, Y.; Zou, H.; Yan, X. Display of receptor-binding domain of SARS-CoV-2 Spike protein variants on the *Saccharomyces cerevisiae* cell surface.

Frontiers in Immunology **2022**, *13*, doi:10.3389/fimmu.2022.935573.
28. Zhao, S.; Guo, D.; Zhu, Q.; Dou, W.; Guan, W. Display of Microbial Glucose Dehydrogenase and Cholesterol Oxidase on the Yeast Cell Surface for the Detection of Blood Biochemical Parameters. *Biosensors* **2020**, *11*, doi:10.3390/bios11010013.
29. Liu, Z.; Wu, J.; Ma, Y.; Hao, L.; Liang, Z.; Ma, J.; Ke, H.; Li, Y.; Cao, J. Protective immunity against CyHV-3 infection via different prime-boost vaccination regimens using CyHV-3 ORF131-based DNA/protein subunit vaccines in carp *Cyprinus carpio* var. Jian. *Fish & Shellfish Immunology* **2020**, *98*, 342-353, doi:10.1016/j.fsi.2020.01.034.
30. Jia, L.G.; Wang, C.; Wu, Y.F.; Ma, X.Y.; Zhang, X.; Chu, X.X.; Lu, F.P.; Liu, Y.H. Production of L-α-Glycerylphosphorylcholine from Oil Refining Waste Using a Novel Cold-Active Phospholipase B from *Bacillus velezensis*. *Acs Sustainable Chemistry & Engineering* **2021**, *9*, 13337-13346, doi:10.1021/acssuschemeng.1c04767.
31. Guo, E.P.; Zhao, L.; Li, Z.Y.; Chen, L.; Li, J.W.; Lu, F.P.; Wang, F.H.; Lu, K.; Liu, Y.H. Biodegradation of bisphenol A by a *Pichia pastoris* whole-cell biocatalyst with overexpression of laccase from *Bacillus pumilus* and investigation of its potential degradation pathways. *Journal of Hazardous Materials* **2024**, *474*, doi:10.1016/j.jhazmat.2024.134779.
32. Gao, M.; Zhou, Y.; Yan, J.; Zhu, L.; Li, Z.; Hu, X.; Zhan, X. Efficient precious metal Rh(III) adsorption by waste *P. pastoris* and *P. pastoris* surface display from high-density culture. *Journal of Hazardous Materials* **2022**, *427*, doi:10.1016/j.jhazmat.2021.128140.
33. Sena, R.O.; Carneiro, C.; Moura, M.V.H.; Brêda, G.C.; Pinto, M.C.C.; Fé, L.X.S.G.M.; Fernandez-Lafuente, R.; Manoel, E.A.; Almeida, R.V.; Freire, D.M.G.; et al. Application of *Rhizomucor miehei* lipase-displaying *Pichia pastoris* whole cell for biodiesel production using agro-industrial residuals as substrate. *International Journal of Biological Macromolecules* **2021**, *189*, 734-743, doi:10.1016/j.ijbiomac.2021.08.173.
34. Inokuma, K.; Kurono, H.; den Haan, R.; van Zyl, W.H.; Hasunuma, T.; Kondo, A. Novel strategy for anchorage position control of GPI-attached proteins in the yeast cell wall using different GPI-anchoring domains. *Metabolic Engineering* **2020**, *57*, 110-117, doi:10.1016/j.ymben.2019.11.004.
35. Chen, Z.; Wang, Y.; Cheng, Y.; Wang, X.; Tong, S.; Yang, H.; Wang, Z. Efficient biodegradation of highly crystallized polyethylene terephthalate through cell surface display of bacterial PETase. *Science of The Total Environment* **2020**, *709*, doi:10.1016/j.scitotenv.2019.136138.
